# Can I Influence You? Development of a Scale to Measure Perceived Persuasiveness and Two Studies Showing the Use of the Scale

**DOI:** 10.3389/frai.2019.00024

**Published:** 2019-11-21

**Authors:** Rosemary J. Thomas, Judith Masthoff, Nir Oren

**Affiliations:** ^1^Computing Science, University of Aberdeen, Aberdeen, United Kingdom; ^2^Institute of Global Food Security, Queen's University Belfast, Belfast, United Kingdom; ^3^Computing Science, Utrecht University, Utrecht, Netherlands

**Keywords:** perceived persuasiveness, scale development, behavior change, message type, argumentation schemes

## Abstract

In this paper, we develop and validate a scale to measure the perceived persuasiveness of messages to be used in digital behavior interventions. A literature review is conducted to inspire the initial scale items. The scale is developed using Exploratory and Confirmatory Factor Analysis on the data from a study with 249 ratings of healthy eating messages. The construct validity of the scale is established using ratings of 573 email security messages. Using the data from the two studies, we also show the usefulness of the scale by analyzing the perceived persuasiveness of different message types on the developed scale factors in both the healthy eating and email security domains. The results of our studies also show that the persuasiveness of message types is domain dependent and that when studying the persuasiveness of message types, the finer-grained argumentation schemes need to be considered and not just Cialdini's principles.

## 1. Introduction

Many behavior change interventions have been developed for a wide variety of domains. For example, “Fit4Life” (Purpura et al., 2011) promotes healthy weight management, the ASICA application (Smith et al., 2016) reminds skin-cancer patients to self-examine their skin, the SUPERHUB application (Wells et al., 2014) motivates sustainable travel, while “Portia” (Mazzotta et al., 2007) and “Daphne” (Grasso et al., 2000) encourage healthy eating habits.

Clearly, it is important to measure the effectiveness of such persuasive interventions. However, it is often difficult to measure actual persuasiveness (O'Keefe, 2018). Perhaps the primary three reasons for such difficulties are as follows. First, measuring actual persuasiveness tends to require more time and effort from participants and additional resources. For example, to measure the persuasiveness of a healthy eating intervention, participants may need to provide detailed diaries of their food intake, which are cumbersome and often unreliable (Cook et al., 2000), and may require the provisioning of scales to participants. Also, when studying many experimental conditions, it may be hard to obtain sufficient participants willing to spend the necessary time [e.g., to measure actual persuasiveness of reminders in (Smith et al., 2016) would have required a large number of skin cancer patients]. Second, it is hard to measure actual persuasiveness due to confounding factors. For example, when measuring the persuasiveness of a sustainable transport application, other factors such as the weather may influence people's behavior. Third, there may be ethical issues which make it hard to measure actual persuasiveness. For example, if one wanted to investigate the persuasive effects of different message types to get learners to study more, it may be deemed unethical to do this in a real class room, as learners in the control condition may be seen to be disadvantaged. Purpura et al. (2011) illustrates some of the ethical problems while using persuasive technologies in behavior change interventions.

Because of these difficulties in measuring actual persuasiveness, *perceived* persuasiveness is often used as an approximation of, or the initial step in the measurement of, actual persuasiveness (see Table 1 for example studies that used perceived persuasiveness). Perceived persuasiveness may include multiple factors. For example, perceived effectiveness in changing somebody's attitudes may be different from perceived effectiveness in changing behavior. We would like a reliable scale that incorporates multiple factors as sub-scales, with each sub-scale consisting of multiple items. Such a scale does not yet exist, and researchers have so far had to use their own measures without proper validation.

**Table 1:** Scale items related to measuring perceived persuasiveness, the measurement scale used for each item, and the number of measurement points.

**References**	**Number**	**Scale items**	**Scale measurement**	**Points**
Anagnostopoulou et al. (2017)		The [System] would	Strongly disagree to strongly agree	7
	1	Influence me		
	2	be convincing		
	3	be personally relevant for me		
	4	make me [target behavior]		
Thomas et al. (2017)	5	Motivational	Not very motivating to very motivating	5
	6	Appropriateness	Very inappropriate to very appropriate	
	7	Effectiveness	Very ineffective to very effective	
	8	Convincing	Very unconvincing to very convincing	
Busch et al. (2016)	9	I find this feature useful	Strongly disagree to strongly agree	7
	10	I enjoy using this feature		
		This feature would		
	11	make me more aware of [policy]		
	12	have a positive influence on my attitude toward [policy]		
	13	lead me to comply with [policy].		
Oduor and Oinas-Kukkonen (2017)		The system provides	Strongly disagree to strongly agree	7
	14	trustworthy content		
	15	believable content		
	16	accurate content		
	17	professional information		
Chang et al. (2018)		On average, [communications] are	Strongly disagree to strongly agree	5
	18	persuasive		
	19	compelling		
	20	logical		
	21	plausible		
Zhao et al. (2011)		[Communication] that is	Strongly disagree to strongly agree	5
	22	believable		
	23	convincing		
	24	important to me		
Orji (2014); Orji et al. (2014)		The system would	Strongly disagree to strongly agree	7
	25	influence me		
	26	be convincing		
	27	be personally relevant for me		
	28	make me reconsider my [behavior]		
Zhang et al. (2014)		[Communications] were	Strongly disagree to strongly agree	7
	29	convincing		
	30	persuasive		
	31	strong		
	32	good		
	33	trustworthy		
	34	reliable		
Kaptein et al. (2009)*^a^*		Susceptibility authority:	Totally disagree to Totally agree	7
	35	I always follow advice from my general practitioner.		
	36	When a professor tells me something I tend to believe it is true.		
		Susceptibility consensus:		
	37	If someone from my social network notifies me about a good book, I tend to read it.		
	38	When I am in a new situation I look at others to see what I should do.		
		Susceptibility liking:		
	39	I accept advice from my social network.		
	40	When I like someone, I am more inclined to believe him or her.		
Busch et al. (2013)	41	Before I do something, I want to know how other people have done it, so I can feel more safe.	Fully agree to Fully disagree	9
	42	It is important to me to know what other people are doing.		
	43	I trust information better where the source is specified.		
	44	It is important for me to be precisely informed about things that I need to do, before I do them.		
Hammer et al. (2016)	45	[Communications were]	Not polite to Very polite Not persuasive at all to Very persuasive	7
Hossain and Saini (2014)	46	The [communication] is	Truthful to Not truthful Unbelievable to Believable Not deceptive to Deceptive	8
	47	The [communicator] is	Sincere to Insincere Honest to Dishonest Not manipulative to Manipulative Not pushy to Pushy	
Meschtscherjakov et al. (2016)		This system	Completely disagree to completely agree	7
	48	makes people change their behavior		
	49	has the potential to influence people		
	50	gives the behavior of its users a new direction		
	51	is exactly what I need to change my attitude		
	52	does not cause a change in behavior with me		
	53	causes me to do some things differently		
	54	Thanks to the system I reach my goals.		
	55	I will use this system as often as possible.		
	56	With the help of the system, I will behave differently in the future.		
Koch and Zerbac (2013)	57	I had the feeling that [communicator] wanted to convince the reader of [communicator]'s standpoint	I do not agree at all to I fully agree	5
	58	[Communicator] wanted to convince me of [communicator]'s views		
MacKenzie and Lutz (1989)	59	Attitude: The [communicator/communication] is	Good to bad Pleasant to Unpleasant Favorable to Unfavorable	7
	60	Credibility: The [communicator/communication] is	Convincing to Unconvincing Believable to Unbelievable Biased to Unbiased	

a *This is a sample. They also present items related to the susceptibility to the other Cialdini principles*.

Therefore, this paper describes the process for developing a reliable and validated multi-item, multi-subscale scale to measure perceived persuasiveness. In addition, the data collected will be used to show the usefulness of the scale by analyzing the impact of different persuasive message types on the developed scale factors.

## 2. Literature Review

To inspire the scale items and show the need for scale development, we first investigated how researchers measured perceived persuasiveness by examining the scale items and respective measurements they used in published user studies. We performed a semi-structured literature review, searching in Scopus from the period 2014 to 2018 across disciplines. At first we performed a narrow search using the following search query:

“*scales development” AND studies AND persuasion*.

However, this produced very few search results. Later, we modified the search query to the following:

persuasion AND (experiments OR studies)

to get a broader range of articles. We also searched in the Proceedings of the “International Conference on Persuasive Technology” for the period from 2013 to 2018. We were looking for user studies that developed or used a scale to measure perceived persuasiveness. The search resulted in 12 papers, including 2 from outside computer science from marketing and communications (Koch and Zerbac, 2013; Zhang et al., 2014). Ham et al. (2015) and O'Keefe (2018) appeared in the initial search results but were excluded as they contained meta-reviews rather than original studies. Three papers were added to the results through snowballing, given these specifically addressed perceived persuasiveness scales:

- Kaptein et al. (2009) cited in Busch et al. (2013).
- MacKenzie and Lutz (1989) cited in Ham et al. (2015).
- Zhao et al. (2011) cited in O'Keefe (2018).

The results of the literature search are shown in Table 1, which lists 60 scale items and their measurements based on studies reported in these 15 papers^1^.

Unfortunately, most studies do not report on the scale construction, reliability or validation. The exceptions are Kaptein et al. (2009) and Busch et al. (2013). However, Kaptein et al. (2009)'s scale really measures the susceptibility of participants to certain Cialdini's principles of persuasion (such as liking and authority) (Cialdini, 2009), rather than the persuasion of the messages themselves. Similarly, Busch et al. (2013) aims to measure the persuasibility of participants by certain persuasive strategies (such as social comparison and rewards).

We reduced the 60 items listed in Table 1 in two steps. First, we removed duplicates and merged highly similar items. Next, we transformed items that were not yet related to a message where possible (items 9, 11–13, 35–36). For instance, item 11 “This feature would make me more aware of [policy]” was changed into “This message makes me more aware of my behavior,” and item 35 “I always follow advice from my general practitioner” was changed into “I will follow this message.” Finally, we removed items for which this was not possible (e.g., items 37–44 that measure a person's susceptibility, and items such as 10, 55). This reduced the list to the 30 items used for the initial scale development as shown in Table 2, which also shows which original items these were derived from.

**Table 2:** Scale items developed used in Study 1.

**Scale items**		**Inspired from / Similar to**
This message is	influencing.^®^	1,25
	convincing. ^®^	2,8,23,26,29,57,58
	personally relevant.^®^	3,24,27
	motivating. ^®^	5
	appropriate.	6
	credible.	21,31,60
	encouraging. ^®^	7,18,30,45
	inappropriate.^*^	N/A
	effective.^®^	7
	useful.^®^	9,17,32
	believable. ^®^	15,22,34,46,47
	ineffective.^*^	N/A
	accurate.	16,17,20
	trustworthy.	14,33,46,47
	exactly what I need to help reach my goals.	54
	exactly what I need to change my attitude.	49,51
	exactly what I need to change my behavior.	51
This message	makes me more aware of my behavior.^®^	11
	leads me to comply with behavior expectations.^®^	13,19
	will cause changes in my behavior.	4
	has a positive influence on my attitude.^®^	12
	has the potential to change user behavior.	48
	has the potential to influence user behavior.	49
	has the potential to inspire users.	50
	causes a change in my behavior.	4,52
	causes me to make some changes in my behavior.	4,53
I	will follow this message.^®^	13,19,35
	consider this message.	28
	accept this message.	39
	believe this message is true.^®^	36
After viewing this message, I will make	some behavior change in the future. changes in my attitude.	56 49,51
Please click the second option from the	right.^*^ left.^*^	N/A N/A

* *act as validation check*.

® *cross loaded on different factors*.

A limitation of our systematic literature review is that it was mainly restricted to papers published in the period 2014–2018^2^. Additionally, it is possible for a systematic review to miss papers due to the search terms used or the limitation of searching abstracts, titles, and keywords. Some other papers related to measuring persuasiveness were found after the review was completed, most noticeably (Feltham, 1994; Allen et al., 2000; Lehto et al., 2012; Popova et al., 2014; Jasek et al., 2015; Yzer et al., 2015; McLean et al., 2016). We will discuss how the scales developed in this paper relate to this other work in our discussion section.

## 3. Study Design

### 3.1. Study 1: Development of a Perceived Persuasiveness Scale

We conducted a study to develop a rating scale to measure the “perceived persuasiveness” of messages. The aim was to obtain a scale with good internal consistency, and with at least three items per factor following the advice in MacCallum et al. (1999) to have at least three or four items with high loadings per factor.

#### 3.1.1. Participants

The participants for this study were recruited by sharing the link of the study via social media and mailing lists. The study had four validation questions to check if participants were randomly rating the scales. After removing such participants, a total of 92 participants rated 249 messages.

#### 3.1.2. Procedure

**Figure 1:**
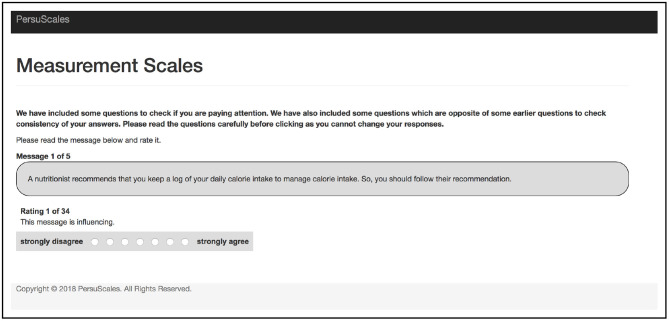
Screenshot of Study 1 showing a message with a scale item to be rated.

Each participant was shown a set of five messages (see Table 4), each promoting healthy eating. These messages were based on different *argumentation schemes*^3^ (Walton et al., 2008) and were produced in another study using a message generation system (Thomas et al., 2018). Each message was rated using 34 scale items (the scale items marked with * act as validation checks) on a 7-point Likert scale that ranges from “strongly disagree” to “strongly agree” (see Table 2 and Figure 1). Finally, participants were given the option to provide feedback.

#### 3.1.3. Research Question and Hypothesis

We were interested in the following research question:

- RQ1: What is a reliable scale to measure perceived persuasiveness?

In addition, we wanted to investigate the usefulness of the scale by analyzing whether the different message types had an impact on the ratings of the developed factors. Therefore, we formulated the following hypothesis:

- H1: Perceived persuasiveness of each factor differs for different message types.

### 3.2. Study 2: Validation of the Perceived Persuasiveness Scale

Next, we conducted a study to determine the construct validity of the developed scale. We replicated the scale-testing in the domain of email security using another data set.

#### 3.2.1. Participants

The participants for this study were recruited by sharing the link of the study via social media and mailing lists. After removing the invalid participants (as before), a total of 134 participants rated 573 messages.

#### 3.2.2. Procedure

**Figure 2:**
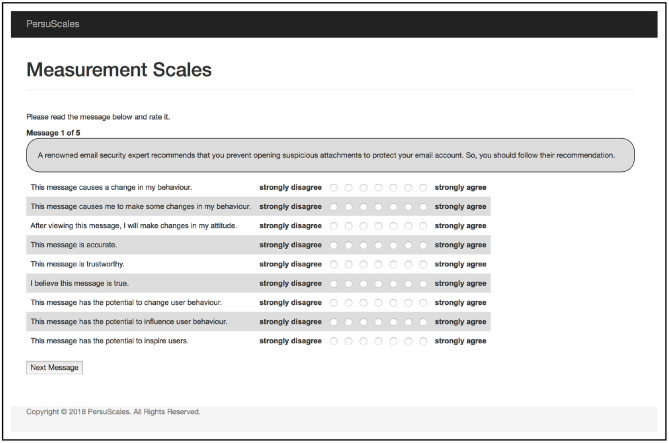
Screenshot of Study 2 showing message with the scale items to be rated.

Each participant was shown a set of five messages (see Table 5) that promote email security, again based on argumentation-schemes. Each message was rated using the scale (see Table 6 and Figure 2) that resulted from Study 1. Finally, participants were given the option to provide feedback.

#### 3.2.3. Research Question and Hypotheses

We were interested in the following research question:

- RQ2: How valid is the developed perceived persuasiveness scale?

Our first study: Development of a Perceived Persuasiveness Scale resulted in a scale with three factors for measuring perceived persuasiveness: Effectiveness, Quality, and Capability (see section 4.1). We wanted to investigate the usefulness of this scale by analyzing whether the message types differed on these three developed factors. Therefore, we formulated the following hypotheses:

- H2: The perceived persuasiveness factor Effectiveness differs for different message types.
- H3: The perceived persuasiveness factor Quality differs for different message types.
- H4: The perceived persuasiveness factor Capability differs for different message types.
- H5: Overall perceived persuasiveness^4^ differs for different message types.

## 4. Results

### 4.1. Study 1: Development of a Perceived Persuasiveness Scale

First we checked the Kaiser-Meyer-Olkin Measure of Sampling Adequacy, which was greater than 0.90. According to this measure, values in the 0.90's indicate that the sampling adequacy is “marvelous” (Dziuban and Shirkey, 1980). Next, we investigated the inter-item correlations. For the factor analysis, all the 7-point scale items were considered as ordinal measures. To further filter the items and identify the factors, we conducted an Exploratory Factor Analysis (EFA) using Principal Component Analysis extraction and Varimax rotation with Kaiser Normalization (Howitt and Cramer, 2014). Varimax rotation was used as the matrix was confirmed orthogonal (the Component Correlation Matrix shows that the majority of the correlations was less than 0.5). We obtained three factors (see Table 2). The first factor we named Effectiveness as its items relate to user behavior and attitude changes and attainment of user goals. The second we named Quality as its items relate to characteristics of a message strength such as trustworthiness and appropriateness. The third we named Capability as its items relate to the *potential* for motivating users to change behavior. We removed the 13 items that cross loaded on different factors (see Table 2 with scale items marked ^®^). This resulted in Table 3, which shows the reduced scale items for the three factors. We checked the Cronbach's Alpha of all the items belonging to the three factors separately. It was greater than 0.9 for each of the three factors which indicates “excellent” scale reliability.

**Table 3:** Study 1: Reduced scales items after EFA.

**Factors**	**Scale items**
Effectiveness	This message will cause changes in my behavior.^®^
	This message is exactly what I need to help reach my goals.^®^
	This message is exactly what I need to change my attitude.^®^
	This message is exactly what I need to change my behavior.^®^
	This message causes a change in my behavior.
	This message causes me to make some changes in my behavior.
	After viewing this message, I will make some behavior change in the future.^®^
	After viewing this message, I will make changes in my attitude.
Quality	This message is appropriate.^®^
	This message is credible.^®^
	This message is believable.
	This message is accurate.
	This message is trustworthy.
	I believe this message is true.
	I accept this message.^®^
Capability	This message has the potential to change user behavior.
	This message has the potential to influence user behavior.
	This message has the potential to inspire users.

®* high Standardized Residual Covariances with several other items*.

**Table 4:** Healthy eating messages used in Study 1 with corresponding argumentation schemes.

**Scheme name**	**Message**
Argument from commitment with goal	As you want to eat healthy, you are committed to eating healthy foods. So, you are also committed to shopping carefully and reading the labels as it helps you to eat healthy foods.
Argument from expert opinion with goal	A nutritionist recommends that you keep a log of your daily calorie intake to manage calorie intake. So, you should follow their recommendation.
Argument from position to know with goal	A college football star suggests that you eat a diet high in protein to have more energy. So, you should follow their suggestion.
Argument from sunk cost with action	You have a choice whether or not to eat vegetables with every serving, however, you committed to doing so earlier. So, you should choose to eat vegetables with every serving.
Practical reasoning with goal	If you cut out added sugars, white flours, white rice and soft drinks, it helps you to lose weight. So, you ought to do this.

**Table 5:** Email security messages used in Study 2 with corresponding argumentation schemes.

**Scheme name**	**Message**
Argument from commitment with goal	As you want to keep your computer account safe, you are committed to check whether website links are genuine in emails. So, you are also committed to preview website links in an email application as it helps you to check whether website links are genuine in emails.
Argument from expert opinion with goal	A renowned email security expert recommends that you prevent opening suspicious attachments to protect your email account. So, you should follow their recommendation.
Argument from position to know with goal	A colleague who attended email security training suggests that you verify the logo, header and footer of email newsletters to make sure that they originate from genuine sources. So, you should follow their suggestion.
Argument from sunk cost with action	You have a choice whether or not to be security-conscious when processing email; however, you committed to doing so earlier. So, you should choose to be security-conscious when processing email.
Practical reasoning with goal	If you choose not to provide personal information by responding to emails that threaten to disable account access, it helps you to safeguard your email account. So, you ought to do this.

Next, we conducted Confirmatory Factor Analyses (CFA) to determine the validity of the scale, and to confirm the factors and items by checking the model fit (Hu and Bentler, 1999). Based on these analyses, 8 items were removed due to high Standardized Residual Covariances with several other items which were greater than 0.4. The items removed are the items in Table 3 marked ^®^.

Table 6 shows the resulting scale of 9 items. The final Confirmatory Factor Analysis resulted in the following values for the Tucker-Lewis Index (TLI) = 0.988, Comparative Fit Index (CFI) = 0.993, and Root Mean Square Error of Approximation (RMSEA) = 0.054, when extracting the three factors and their items. A cut off value nearing 0.95 for TLI and CFI (the higher the better) and a cut off value nearing 0.60 for RMSEA (the lower the better) are required to establish that there is an acceptable model fit between the hypothesized model and the observed data (Hu and Bentler, 1999; Schreiber et al., 2006). In the resulting scale, the TLI and CFI are above 0.95 and RMSEA is below 0.60, which shows an acceptable model fit. This answers research question RQ1.

**Table 6:** Study 1: Reduced scale items after CFA.

**Factors**	**Scale items**
Effectiveness	This message will cause changes in my behavior. This message causes me to make some changes in my behavior. After viewing this message, I will make changes in my attitude.
Quality	This message is accurate. This message is trustworthy. I believe this message is true.
Capability	This message has the potential to change user behavior. This message has the potential to influence user behavior. This message has the potential to inspire users.

### 4.2. Study 1: Impact of Message Types on Factors

**Figure 3:**
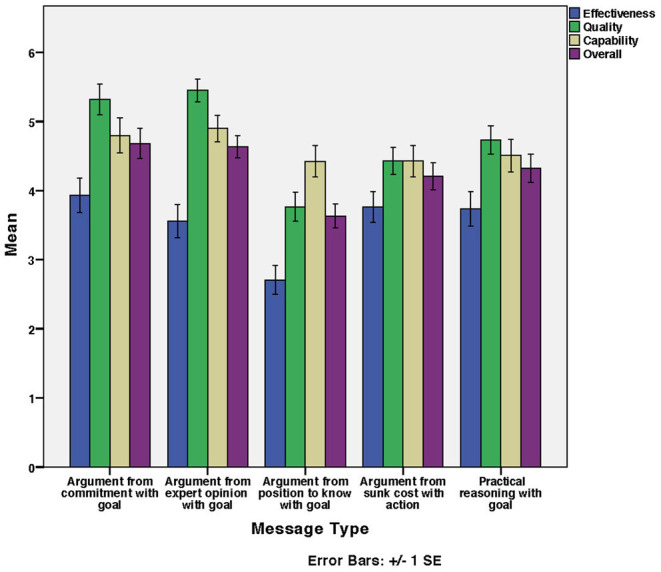
Healthy eating messages: Mean of factors' and overall ratings for developed scale per message type.

Figure 3 shows the mean Effectiveness, Quality, Capability, and Overall perceived persuasiveness of message types used for the healthy eating messages. Overall perceived persuasiveness was calculated as the mean of the factors: Effectiveness, Quality, and Capability.

A one-way repeated measures MANOVA with Effectiveness, Quality, Capability, and Overall perceived persuasiveness as dependent variables and message type as the independent variable provided the results for the analyses given below. To determine the homogeneous subsets, the Ryan-Einot-Gabriel-Welsch Range was selected as a *post-hoc* test since we have more than 3 levels within the independent variable (i.e., the message type).

According to Thomas et al. (2018), the argumentation schemes can be mapped to Cialdini's principles of persuasion.

1. Cialdini's Principle: Commitments and Consistency Argument from commitment with goal Practical reasoning with goal. Argument from sunk cost with action
2. Cialdini's Principle: Authority Argument from expert opinion with goal Argument from position to know with goal.

The study conducted by Thomas et al. (2017) states that Authority was significantly more persuasive, followed by Commitments and Consistency and the other Cialdini principles. We were interested to know whether our findings would be similar. Hence, the analysis will consider both the argumentation schemes and Cialdini's principles when discussing the findings.

#### 4.2.1. Impact of Message Types on Effectiveness

According to Figure 3, Argument from commitment with goal was the highest rated in Effectiveness while Argument from position to know with goal was the lowest. There was a significant effect of message type on Effectiveness [*F*_(4, 244)_ = 4.39, *p* < 0.01]. There was a significant difference between Argument from position to know with goal and the other message types (*p* < 0.05). The rest were non-significant. Table 7 shows the homogeneous subsets. This partially supports the hypothesis (H1) that perceived persuasiveness on each factor differs for different message types.

**Table 7:** Study 1: Homogeneous subsets for Effectiveness, Quality, and Capability.

	**Effectiveness**			**Quality**					**Capability**	
**Message type**		**Mean**			**Mean**					**Mean**
	**N**	**S1**	**S2**	**N**	**S1**	**S2**	**S3**	**S4**	**N**	**S1**
Argument from position to know with goal	52	2.71		52	3.76				52	4.42
Argument from expert opinion with goal	52		3.56	51		4.43			51	4.42
Practical reasoning with goal	48		3.74	48		4.73	4.73		48	4.51
Argument from sunk cost with action	51		3.76	46			5.32	5.32	46	4.80
Argument from commitment with goal	46		3.93	52				5.45	52	4.90

As shown, the two Authority messages had the lowest Effectiveness scores, though the Argument from expert opinion with goal was not rated significantly lower than the Commitments and Consistency messages. We observe that the Effectiveness of all messages was low, below or around the mid-point of the scale. This contradicts the results from Thomas et al. (2017) where Authority and Commitments and Consistency messages were most persuasive, though of course their study only considered overall perceived persuasiveness without using a validated scale.

#### 4.2.2. Impact of Message Types on Quality

According to Figure 3, for healthy eating messages Argument from expert opinion with goal was the highest rated in quality while Argument from position to know with goal was the lowest. There was a significant effect of message type on Quality [*F*_(4, 244)_ = 12.14, *p* < 0.001]. There was a significant difference (*p* < 0.05) between:

1. Argument from position to know with goal and the other message types,
2. Argument from sunk cost with action and the other message types except Practical reasoning with goal,
3. Practical reasoning with goal and the other message types except Argument from Commitment with goal, and
4. Argument from Commitment with goal and the other message types except Argument from expert opinion with goal.

Table 7 shows the homogeneous subsets. This partially supports the hypothesis (H1) that perceived persuasiveness on each factor differs for different message types. However, it should be noted that one Authority message is the worst and one the best on Quality. This may either be caused by attributes of the message itself, or by one of the Authority argumentation schemes resulting in higher quality messages than the other one.

#### 4.2.3. Impact of Message Types on Capability

According to Figure 3, Argument from expert opinion with goal was slightly higher rated in quality compared to the other message types. There was no significant effect of message type on Capability [*F*_(4, 244)_ = 0.98, *p* > 0.05]. Table 7 shows the homogeneous subsets. This does not support the hypothesis (H1) that perceived persuasiveness of each factor differs for different message types. All message types performed equally well on Capability, which was above the midpoint of the scale.

#### 4.2.4. Impact of Message Types on Overall Perceived Persuasiveness

According to Figure 3, Argument from commitment with goal was the highest rated overall while Argument from position to know with goal was the lowest. There was a significant effect of message type on Overall Perceived Persuasiveness [ *F*_(4, 244)_ = 4.98, *p* < 0.01]. Argument from position to know with goal was significantly different from Argument from expert opinion with goal and Argument from commitment with goal (*p* < 0.05). The rest were non-significant. Table 8 shows the homogeneous subsets. This partially supports the hypothesis (H1) that each factor differs on different message types.

**Table 8:** Study 1: Homogeneous subsets for Overall Perceived Persuasiveness.

		**Mean**	
**Message type**	***N***	**Subset 1**	**Subset 2**
Argument from position to know with goal	52	3.63	
Argument from sunk cost with action	51	4.21	4.21
Practical reasoning with goal	48	4.32	4.32
Argument from expert opinion with goal	52		4.63
Argument from commitment with goal	46		4.68

### 4.3. Study 2: Validation of the Perceived Persuasiveness Scale

To determine the construct validity of the developed scale in Study 1 and replicate the scale-testing, we:

1. Used an 80-20 split validation on the original dataset of Study 1. With this specific combination, the developed scale resulted in an acceptable model fit for 80% (TLI = 0.975, CFI = 0.985, RMSEA = 0.081) and 20% of the data (TLI = 0.975, CFI = 0.985, RMSEA = 0.080).
2. Used the dataset obtained from the validation in Study 2. With this dataset, the developed model resulted in an acceptable fit (TLI = 0.984, CFI = 0.990, RMSEA = 0.071).

This answers research question RQ2, validating the scale.

### 4.4. Study 2: Impact of Message Types on Factors

**Figure 4:**
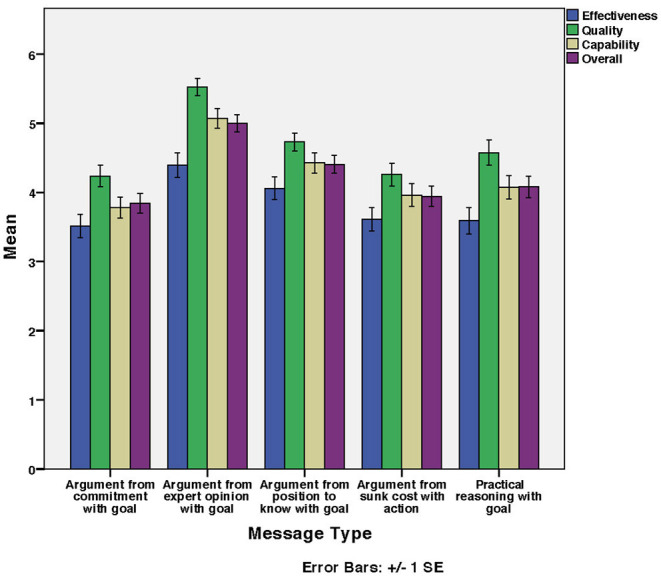
Email security messages: Mean of factors' and overall ratings for developed scale per message type.

Figure 4 shows the mean Effectiveness, Quality, Capability, and Overall perceived persuasiveness of message types used for email security messages. As before, the Overall perceived persuasiveness was calculated as the mean of the factors Effectiveness, Quality, and Capability.

A one-way repeated measures MANOVA with Effectiveness, Quality, Capability, and Overall perceived persuasiveness as dependent variables and message type as the independent variable provided the results for the analyses given below. To determine the homogeneous subsets, the Ryan-Einot-Gabriel-Welsch Range was selected as *post-hoc* test since we have more than 3 levels within the independent variable (i.e., message type).

#### 4.4.1. Impact of Message Types on Effectiveness

According to Figure 4, Argument from expert opinion with goal was the highest rated in Effectiveness while Argument from commitment with goal was the lowest. There was a significant effect of message type on Effectiveness [*F*_(4, 568)_ = 4.77, *p* < 0.01]. Argument from commitment with goal was significantly different from Argument from position to know with goal and Argument from expert opinion with goal (*p* < 0.05). The rest were non-significant. Table 9 shows the homogeneous subsets. This partly supports hypothesis H2, namely that perceived persuasiveness in terms of Effectiveness differs for different message types.

**Table 9:** Study 2: Homogeneous subsets for Effectiveness, Quality, and Capability.

	**Effectiveness**			**Quality**			**Capability**			
**Message type**		**Mean**			**Mean**			**Mean**		
	**N**	**S1**	**S2**	**N**	**S1**	**S2**	**N**	**S1**	**S2**	**S3**
Argument from commitment with goal	115	3.51		115	4.24		115	3.78		
Practical reasoning with goal	111	3.59		115	4.26		115	3.96	3.96	
Argument from sunk cost with action	115	3.61		111	4.58		111	4.08	4.08	
Argument from position to know with goal	117	4.06	4.06	117	4.73		117		4.43	
Argument from expert opinion with goal	115		4.40	115		5.52	115			5.07

The subsets show that Authority messages in the email security domain performed better on Effectiveness than Commitments and Consistency messages. This is in line with the findings of the study by Thomas et al. (2017) and contradicts what was found in Study 1 for the healthy eating messages.

#### 4.4.2. Impact of Message Types on Quality

According to Figure 4, Argument from expert opinion with goal was the highest rated in Quality while Argument from commitment with goal was the lowest. There was a significant effect of message type on Quality [*F*_(4, 568)_ = 11.97, *p* < 0.001]. Argument from expert opinion with goal was significantly different from the other message types (*p* < 0.05). The rest were non-significant. Table 9 shows the homogeneous subsets. This partially supports hypothesis H3, namely that perceived persuasiveness in terms of Quality differs for different message types.

We observe that Argument from expert opinion with goal was rated significantly higher than the other message types and that the other Authority message had the second highest mean. Therefore, in the domain of email security, we can conclude that principle of Authority seems most persuasive when considering Quality. We note that Argument from expert opinion with goal performed best on Quality in both Studies, so this argumentation scheme seems to result in good quality messages. In contrast, Argument from position to know with goal did not do as well in the healthy eating domain. It is possible that this is a domain effect, with people trusting people with experience more in the cyber-security domain than in the healthy eating domain. We will investigate this finding further as future work.

#### 4.4.3. Impact of Message Types on Capability

According to Figure 4, Argument from expert opinion with goal was the highest rated in Capability while Argument from commitment with goal was the lowest. There was a significant effect of message type on Capability [*F*_(4, 568)_ = 10.84, *p* < 0.001]. There was significant difference (*p* < 0.05) between

1. Argument from expert opinion with goal and the other message types.
2. Argument from commitment with goal and Argument from position to know with goal.

There were no significant differences between Argument from sunk cost with action and Practical reasoning with goal. Table 9 shows the homogeneous subsets. This partially supports hypothesis H4 that perceived persuasiveness in terms of Capability differs for different message types.

We observe that Argument from expert opinion with goal was rated significantly higher than other message types, and that the other Authority message was rated second highest. Therefore, we can conclude that the principle of Authority was also most persuasive when considering Capability. Again, we can see domain effects in this finding, with Argument from position to know performing better compared to other message types in the email security domain.

#### 4.4.4. Impact of Message Types on Overall Perceived Persuasiveness

According to Figure 4, Argument from expert opinion with goal was the highest rated in overall perceived persuasiveness whilst Argument from commitment with goal was the lowest. There was a significant effect of message type on overall perceived persuasiveness [*F*_(4, 568)_ = 11.24, *p* < 0.001]. Table 10 shows the homogeneous subsets. This partially supports hypothesis H5 that the overall perceived persuasiveness differs for different message types.

**Table 10:** Study 2: Homogeneous subsets for overall perceived persuasiveness.

		**Mean**		
**Message type**	***N***	**Subset 1**	**Subset 2**	**Subset 3**
Argument from commitment with goal	115	3.84		
Argument from sunk cost with action	115	3.94	3.94	
Practical reasoning with goal	111	4.08	4.08	
Argument from position to know with goal	117		4.41	
Argument from expert opinion with goal	115			5.00

The overall perceived persuasiveness results are similar to those for “Impact of message type on Capability”; again overall Authority messages performed well, and better than in the healthy eating domain.

## 5. Discussion

Our studies resulted in a validated perceived persuasiveness scale as well as insights into the perceived persuasiveness of different message types.

### 5.1. The Perceived Persuasiveness Scale

Regarding the scale, as mentioned in the limitations of the systematic literature review, there are some other papers that proposed persuasiveness scales that were not part of the review. The uptake of these scales has been limited as judged by them not having been used in the reviewed papers. However, it is interesting to see how these scales compare to the one developed in this paper, and to consider what overlap/differences there are.

First, Feltham (1994) developed and validated a Persuasive Discourse Inventory (PDI) scale based on Aristotle's three types of persuasion: ethos, pathos, and logos (see Table 11). Ethos relates to the credibility of the message source, pathos to the message's affective appeal, and logos to its rational appeal. To validate the PDI scale, they mainly considered Cronbach's alpha rather than conducting a factor analysis as was done in this paper. Their results suggest that there may be cross-loadings between their scale factors as they found a positive correlation between Logos and Ethos. They also did not consider whether the scale performed well across domains, as their reassessment was conducted in a very similar domain. Regarding the scale content, the scale developed in this paper has more items that directly inquire into a message's perceived persuasiveness rather than the emotional and logical elements present in the messages, though Ethos, Logos, and Pathos still play a role. Several Ethos related items were included in our initial scale development items, namely trustworthy, believable, and credible. One of these items (cf. trustworthy) has remained in the validated scale as part of the Quality factor. The “accurate” item that is part of the Quality factor can be interpreted as on the overlap between Ethos and Logos, as it on the one hand gives a sense of being reliable, and on the other of being based on facts/rational/logical. Regarding Pathos, the item “This message has the potential to inspire users” in the Capability factor is clearly related to Pathos (as was the item “motivating” that did not make it into the final scale).

**Table 11:** Persuasive Discourse Inventory (Feltham, 1994).

**Ethos sale items: Ethos = E1+E2+E3+E4+E5 (range: 5-35)**
E1) unbelievable / believable
E2) not credible / credible
E3) not trustworthy / trustworthy
E4) unreliable / reliable
E5) undependable / dependable
Logos scale items: Logos = LI+12+L3+14+L5 (range 5-35)
L1) not rational / rational
L2) not informative / informative
L3) does not deal with facts / deals with facts
L4) not knowledgeable / knowledgeable
L5) not logical / logical
Pathos scale items: Pathos = PI+P2+P3+P4+P5+P6+P7 (range: 7-49)
P1) does not affect my feelings / affects my feelings
P2) does not touch me emotionally / touches me emotionally
P3) is not stimulating / is stimulating
P4) does not reach out to me / reaches out to me
P5) is not stirring / is stirring
P6) is not moving / is moving
P7) is not exciting / is exciting

Second, Lehto et al. (2012) developed a model with factors that predict perceived persuasiveness, and as part of this also considered the internal consistency of items to measure these factors. Several of their factors (e.g., dialogue support, design aesthetics) are not directly about persuasive messages *per se* but rather about the overarching behavioral intervention system they were studying. The aim of their work was not to develop a scale, so they did not try to develop factors that are independent of each other, but were mainly interested in how the factors related to each other. In fact, despite finding adequate internal consistency, they found quite a lot of cross-loadings, with items from one factor loading above 0.5 on other factors as well. Their validation was only in the health domain, and many of their questions specifically related to their intervention (e.g., a primary task support item “NIV provides me with a means to lose weight,” a dialogue support item “NIV provides me with appropriate counseling,” a perceived credibility item “NIV is made by health professionals”). So, this work did not result in a multi independent factors scale that can be used in multiple domains, like the scale developed in this paper. Considering the factors they considered, Perceived Credibility overlaps with the Quality factor in our scale (cf. trustworthy). Primary Task support is related to the Effectiveness factor in our scale (e.g., “helps me change [my behavior]” is related to “causes a change in my behavior”). Their Perceived Persuasiveness factor has some relation to our Capability factor (e.g., compare “has an influence on me” and “has the potential to influence user behavior,” “makes me reconsider [my behavior],” and “has the potential to change user behavior”).

Third, Allen et al. (2000) compared the persuasiveness of statistical and narrative evidence in a message, and produced two scales to perform this study: a Credibility scale (measuring the extent to which one trusts the message writer) and an Attitude scale (measuring the extent to which one accepts the message's conclusion). They checked that each scale only contained one factor, and that each scale was internally consistent (in terms of Cronbach's alpha). They did not, however, consider whether items from one scale cross-loaded onto the other scale (e.g. the items “I think the writer is wrong” from the Attitude scale and “the writer is dishonest” from the Credibility scale seem related, so cross-loadings may well occur). They also did not remove an item with low factor loading (“the writing style is dynamic,” loading 0.40) from the Credibility scale, which may indicate a poor scale structure (MacCallum et al., 1999). Their scales only measure some aspects of persuasiveness; for example, they do not measure the message's potential to inspire, or to cause behavior change.

Fourth, Popova et al. (2014), Jasek et al. (2015), and Yzer et al. (2015) used multi-item scales, but without a development phase. Popova et al. (2014) used five items (convincing-unconvincing, effective-ineffective, believable-unbelievable, realistic-unrealistic, and memorable-not memorable), Jasek et al. (2015) 13 (boring, confusing, convincing, difficult to watch, informative, made me want to quit smoking, made me want to smoke, made me stop and think, meaningful to me, memorable, powerful, ridiculous, terrible), and Yzer et al. (2015) 7 (convincing, believable, memorable, good, pleasant, positive, for someone like me). There is considerable overlap between these items and the ones we used for the scale development, though there are some items in these papers that seem more related to usability (e.g., “confusing”) and some more related to feelings (e.g., “pleasant,” “terrible”).

Fifth, McLean et al. (2016) developed a scale from 13 items for measuring the persuasiveness of messages to reduce stigma about bulimia. They only performed an exploratory factor analysis (using ratings of only 10 messages), so no real validation. Their scale has two factors; one they describe as convincingness and the other as likelihood of changing attitudes toward bulimia. The first factor includes items such as “believable” and “convincing,” which were part of our initial items for scale development and are related to the Quality factor in our scale. The second factor is related to the Capability factor of our scale.

In summary, the scale developed in this paper is unique in that it was developed from a large set of items covering a wide range of aspects of persuasiveness, was developed and validated across two domains, and has been shown to consist of three independent factors, with good internal consistency. The comparison of scale content with the content of other scales shows that the scale also provides reasonable coverage of concepts deemed important in the literature (for example, some aspects of Ethos, Pathos, and Logos are present).

### 5.2. Persuasiveness of Message Types

As a side effect of our studies, we also gained insights into the persuasiveness of message types. There have been several other papers investigating this, though these studies have only investigated the impact of Cialdini's principles and not the finer-grained argumentation schemes. For instance, Orji et al. (2015) and Thomas et al. (2017) investigated the persuasiveness of Cialdini's principles for healthy eating, Smith et al. (2016) for reminders to cancer patients, Ciocarlan et al. (2018) for encouraging small acts of kindness, and Oyibo et al. (2017) in general without mentioning specific domains.

Thomas et al. (2017) found that Authority messages were most persuasive and Liking least persuasive. Orji et al. (2015) found that Commitment and Reciprocity were the most persuasive over all ages and gender, whereas Consensus and Scarcity were the least persuasive. They found that females responded better to Reciprocity, Commitment, and Consensus messages than males. They also observed that adults responded better to Commitment than younger adults, and younger adults responded better to Scarcity than adults. Smith et al. (2016) observed that Authority and Liking were the most popular for the first reminder, and there was a preference for using Scarcity and Commitment for the second reminder. Ciocarlan et al. (2018) found that the Scarcity message worked best. Oyibo et al. (2017) observed that their participants were more susceptible to Authority, Consensus, and Liking.

The conflicting results of these studies can have several causes. Firstly, the studies were conducted in different domains. Our studies in this paper have shown that the persuasiveness of message types is in fact domain dependent. For example, we found in the Healthy Eating domain that some of the Authority-linked argumentation schemes scored badly on Effectiveness, and one of them was also worst on persuasiveness overall, whilst in the Email Security domain Authority-linked argumentation schemes scored best. Secondly, the studies used very different (and not validated) ways of measuring persuasiveness. So, it would be interesting to repeat all of these studies in a variety of domains using the scale developed in this paper. Thirdly, these studies did not consider the finer-grained argumentation schemes, but only Cialdini's principles. It is possible that, for example, the Authority messages used in one study followed a different argumentation scheme (within the Authority set) than those in another study. Finally, in contrast to our studies, none of these papers considered the individual factors of persuasiveness, but only considered persuasiveness as a whole. Our studies show that it is possible for a message type to score badly on one dimension on persuasiveness whilst scoring well on the others.

In summary, the most important results in this paper regarding the persuasiveness of message types are that (1) this persuasiveness is domain dependent, (2) investigating the finer-grained argumentation schemes matters as different results can be obtained for different argumentation schemes that are linked to the same Cialdini's principles, and (3) investigating the different factor of persuasiveness matters as different results can be obtained for the different factors.

## 6. Conclusions

In this paper, we developed and validated a perceived persuasiveness scale to be used when conducting studies on digital behavior interventions. We conducted two studies in different domains to develop and validate this scale, namely in the healthy eating domain and the email security domain. The validated scale has 3 factors (Effectiveness, Quality, and Capability) and 9 scale items as illustrated in Table 6. We also discussed how this scale relates to and extends on earlier work on persuasiveness scales.

In addition to developing a scale, and to show its usefulness, we analyzed the impact of message types on the different developed scale factors. We found that message type significantly impacts on Effectiveness, Quality, and overall perceived persuasiveness in studies in both the healthy eating and email security domains. We also found a significant impact of message type on Capability in the email security domain. The three factors (as shown in the validation) measure different aspects of perceived persuasiveness. One example where this can also be seen is for the Argument from expert opinion with goal message type, which performs relatively badly on Effectiveness in the healthy eating domain but well on Quality in that domain. The persuasiveness of messages is clearly domain dependent. Additionally, our studies show that it is worthwhile to investigate the finer-grained argumentation schemes rather than just Cialdini's principles. We discussed related work on measuring the persuasiveness of message types and explained the conflicting findings in those studies.

As shown in our literature review, researchers working on digital behavior interventions tend to use their own scales, without proper validation of those scales, to investigate perceived persuasiveness. The validated scale developed in this paper can be used to improve such studies and will make it easier to compare the results of different studies and in different domains. We plan to use the scale to study the impact of message personalization across domains.

The work presented in this paper has several limitations. Firstly, we validated the scale in two domains (healthy eating and email security), and this validation needs to be extended to more domains. Secondly, the scale reliability needs to be verified. To investigate this, we need to perform a test-retest experiment in which participants complete the same scale on the same items twice, with an interval of several days between the two measurements. This also would need to be done in multiple domains. Thirdly, we need to repeat our studies into the impact message types with more messages and in more domains.

## Data Availability Statement

The datasets generated for this study are available on request to the corresponding author.

## Ethics Statement

The studies involving human participants were reviewed and approved by CoPs ethics committee University of Aberdeen. The patients/participants provided their written informed consent to participate in this study.

## Author Contributions

RT and JM contributed to the conception and design of the study. RT implemented the study, performed the statistical analysis, and wrote the first draft of the manuscript. All authors wrote sections of the manuscript, contributed to manuscript revision, read, and approved the submitted version.

### Conflict of Interest

The authors declare that the research was conducted in the absence of any commercial or financial relationships that could be construed as a potential conflict of interest. The reviewer KS declared a past collaboration with one of the authors JM to the handling editor.

## Footnotes

^1^Many of these papers contained additional items; these were normally not related to measuring persuasiveness.

^2^In this period much research on persuasive technology has taken place, as evidenced by 7,410 papers being found for “persuasive technology” in Google Scholar.

^3^Argumentation schemes are stereotypical patterns of reasoning.

^4^Overall perceived persuasiveness was calculated as the mean of the factors: Effectiveness, Quality, and Capability.
